# Long-term outcome in COPD patients with pneumonic and nonpneumonic exacerbation: a 6-year prospective follow-up study

**DOI:** 10.1186/cc13238

**Published:** 2014-03-17

**Authors:** EG Grolimund, AK Kutz, MA Alan, BM Mueller, PS Schuetz

**Affiliations:** 1 Kantonsspital Aarau, Switzerland

## Introduction

Predicting long-term outcome in patients surviving a pneumonic or nonpneumonic COPD exacerbation remains challenging. This study investigates the association of clinical parameters and the prognostic blood marker pro-adrenomedullin (proADM) measured upon hospital discharge with 6-year mortality in well-defined cohort of COPD patients.

## Methods

We prospectively followed consecutive COPD patients from a previous Swiss multicenter trial (2006 to 2008) [1] over a 6-year follow- up and investigated all-cause mortality following hospital discharge. Patients and/or treating general practitioners were contacted by telephone interview to assess the vital status of patients. We used Cox regression models and the area under the receiver operating characteristics curve (AUC) to investigate associations of baseline predictors and mortality.

## Results

Overall mortality in the 469 included COPD patients was 55% (95% CI 0.5 to 0.6) with a 14% (95% CI 0.1 to 0.2) mortality incidence rate per year. Patients with pneumonic COPD exacerbation had a more pronounced inflammatory response compared with patients with nonpneumonic exacerbation with regard to levels of initial C-reactive protein levels (median 158 mg/dl vs. 39 mg/dl, *P *< 0.0001), procalctionin (median 0.4 μg/l vs. 0.1 μg/l, *P *< 0.0001) and proADM (median 1.3 nmol/l vs. 0.9 nmol/l, *P *< 0.0001), but long-term survival was similar (HR 1.0, 95% CI 0.8 to 1.2). In univariate regression models, proADM was significantly associated with mortality after 1, 3 and 6 years (HR 16.1 (95% CI 6.9 to 37.7), 10.5 (95% CI 5.7 to 19.6) and 10.4 (95% CI 6.2 to 17.7), respectively). There was no effect modification by type of exacerbation. A model including clinical parameters (age, coronary heart disease, heart failure, diabetes mellitus, chronic renal failure, neoplastic disease, pneumonia, smokers) and proADM showed good discrimination of long-term survivors from nonsurvivors with AUC of 0.74 (95% CI 0.6 to 0.7).

## Conclusion

Clinical parameters and discharge levels of proADM allow accurate long-term prognostication in COPD patients independent of initial type of exacerbation. The focus on the best use of long-term prognostic information to improve patient care and clinical outcomes seems promising/rational.
